# Adaptive electronic relay for smart grid based on self-healing protection

**DOI:** 10.1371/journal.pone.0309966

**Published:** 2024-10-23

**Authors:** M. Nasrallah, Ahmed Abdelaleem, Mohamed A. Ismeil, Hany S. Hussein

**Affiliations:** 1 Electrical Engineering Department, Faculty of Engineering, South Valley University, Qena, Egypt; 2 Electrical Engineering Department, Faculty of Engineering, King Khalid University, Abha, Saudi Arabia; Hanshan Normal University, CHINA

## Abstract

The protection system is crucial for grid stability and safeguarding essential components, including generators, transformers, transmission systems, and power connections. The smart grid system increases the flexibility and complexity of the power system, making fault detection and isolation the primary challenges for the protection system. This paper presents an optimal protection solution using an adaptive electronic relay to enhance reliability and enable self-healing. The proposed protection algorithm quickly detects faults and automatically isolates them from the rest of the healthy system in 25ms. The relay operation algorithm has been validated using MATLAB SIMULINK software. The results confirm the effectiveness of the proposed smart electronic relay in various sections of the smart grid system, including transformers, transmission and distribution

## 1. Introduction

The protection system is integral to safeguarding all network components from damage, thereby saving costs by preventing network downtime. Given the imperative of transitioning to a smart grid, it’s essential to upgrade the protection system from the conventional grid to a smart one. The literature review is divided into three paragraphs to clarify the focus of this paper: protection systems, smart grids, and protection systems in smart grids. Protection Systems: Numerous studies have explored protection systems [1–4]. A novel hybrid genetic algorithm-particle swarm optimization-linear programming (GA-PSO-LP) algorithm for microgrid protection systems is introduced in [1]. In [2], the remote operation of the relay, the third part of the protection system, is proposed. An adaptive overcurrent protection system that automatically adjusts relay settings in response to distributed grid systems, active network management, and islanding operations is presented in [3]. An innovative adaptive directional overcurrent protection system for electric power distribution systems with respect to distributed generation is discussed in [4]. Smart Grids: Several studies have examined smart grids without focusing on protection systems [5–9]. A review and model for smart grids are provided in [5]. A smart transmission and distribution system is proposed in [6]. The impact of smart grids on national grids is discussed in [7]. The performance of connecting electric vehicles to an adjusted smart grid distribution system using an Automatic Variac Transformer (AVT) is presented in [8]. Intelligent power quality management and the integration of renewable energy sources through machine learning and Internet of Things technology are outlined in [9]. Some studies have proposed optimization techniques to achieve optimal values for components used in electrical grid systems, contributing to the goals of the smart grid [10–13]. In [10], modified whale optimization algorithms were introduced to determine the capacitance and inductance per unit length for both single-phase and three-phase systems with varying numbers of bundle conductors. In [11], the flux linkage method was suggested for determining transmission line parameters. In [12], a whale optimization algorithm was employed to determine the parameters of overhead AC transmission lines. In [13], a new optimization method called grey wolf optimization was used to determine the parameters of overhead transmission lines. Protection Systems in Smart Grids: Numerous papers have addressed protection systems within smart grids [14–17]. Protection solutions for smart grids, focusing on overcurrent, transformer, and directional overcurrent protection, are presented in [14]. Techniques to mitigate false tripping are discussed in [15]. A protection scheme for smart grids and microgrids using Dual-Setting Directional Overcurrent Relays (DS-DOCR) is introduced in [16]. The requirements for smart grid protection and the stages of converting traditional systems to smart protection systems are explored in [17]. A multi-layer protection scheme for medium voltage distribution systems is proposed in [18]. Protection system applications in smart distribution grids are presented in [19]. A novel data privacy protection algorithm for distributed smart grid terminals is discussed in [20]. The significance of relays in smart grids is highlighted in [21]. A new protection type based on Busbar Area Protection (BAP) zone is introduced in [22]. Comprehensive lightning protection solutions for smart grids are outlined in [23]. The behavior of Doubly-Fed Induction Generators (DFIG) using PSCAD/EMTDC software is examined in [24] and [25]. An active optical routing assignment algorithm is proposed in [26]. The selectivity and sensitivity of overcurrent relay protection against single-phase faults are discussed in [27]. Traditional relays in the traditional protection system contain the fixed setting parameter. With the increase in demand for energy, electrical power systems have become complex and contain many problems, including the increase in electrical harmonics, which leads to the incorrect operation of protection systems. The primary aim of the protection system is to quickly isolate faulty parts from the healthy system. Therefore, this paper proposes an adaptive electronic relay, which offers a faster response and lower cost compared to electro-mechanical switches. This study focuses on enhancing the reliability and self-healing capabilities of smart systems through a backup protection system that operates after primary protection systems like control systems (automatic crowbar systems). Consequently, the response time must be brief but longer than that of the primary protection systems. To meet the objectives of smart grid systems, the protection system must evolve. It includes five key components: a circuit breaker, trip circuit, instrument transformer, communication channels, and relay, with smart relay technology providing a pathway to advanced protection. The structure of this paper is outlined as follows: the second section illustrates the overall system. The third section introduces an adaptive electronic relay for the smart protection system, detailing the control model designed to achieve the self-healing aims of the smart grid system. The fourth section presents the simulation results of the proposed adaptive electronic relay under three different fault conditions. Finally, the conclusion and future work are discussed.

## 2. System under study

**Fig 1:**
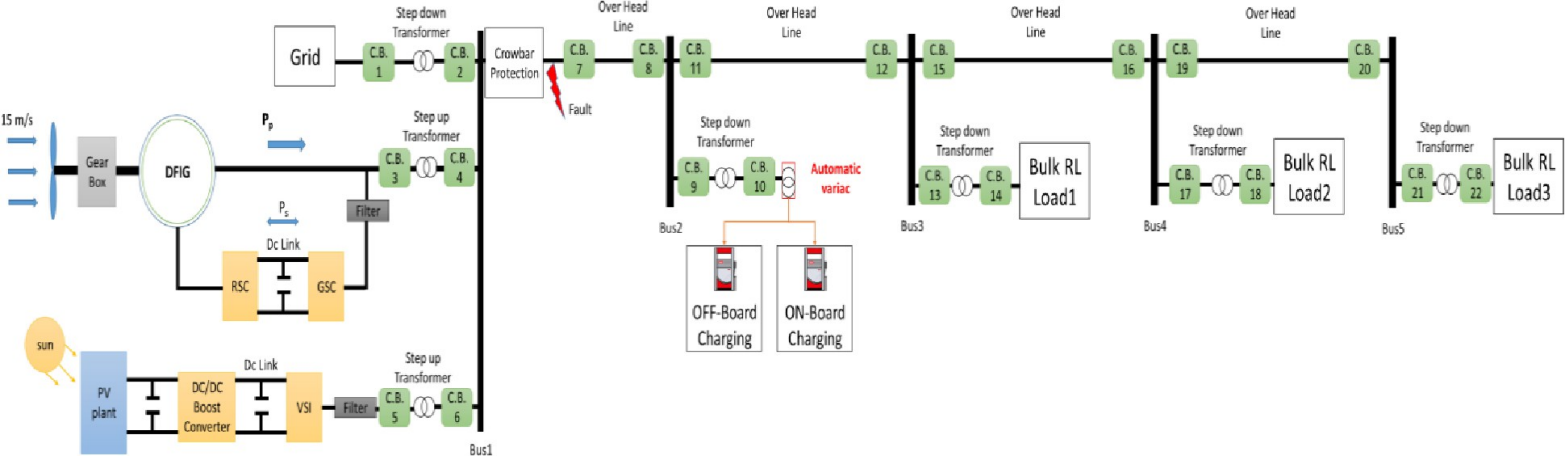
Smart grid system with circuit breakers distributed on the system.

Distributed circuit breakers were added to the smart grid system in [8] to protect each element, as shown in Fig 1. This system was selected as a case study to validate the proposed adaptive electronic relay as part of a smart protection system. The smart system discussed in [8] is termed "smart" because it incorporates renewable energy at the generation stage, an automatic crowbar protection system at the transmission stage, and an EV charging station as a smart load at the load stage. It includes a wind and a PV generation station which are connected to the grid. The grid has a step-down transformer from 33kV to 11kV. The wind generation station features six turbines, each generating 1.5 MW, and uses a step-up transformer from 575V to 11kV. Additionally, a PV generation station was added, consisting of 70 modules in series per string and 300 strings in parallel, collectively generating 3MW, with a step-up transformer from 260V to 11kV. The system is integrated with an automatic controllable crowbar system, which includes resistance equal to 450Ω at the point of common coupling (PCC) at the generation stage. The crowbar’s objective is to mitigate the impact of fault disturbances. The load is distributed across four buses, numbered 2, 3, 4, and 5. Each bus uses a step-down transformer from 11kV to 380V, except for bus number 2, which has an EV charging station. The bulk load on each bus is detailed in Table 1. Circuit breakers are distributed throughout the grid system to protect each element. In this paper, an adaptive electronic relay is employed for grid protection.

**Table 1:** Load data or power demand.

Bus	Type	P(W)
2	Smart load four plugs	90k+90k+50k+50k
3	Bulk load1	3M
4	Bulk load2	3M
5	Bulk load3	2M

## 3. Proposed smart protection system

**Fig 2:**
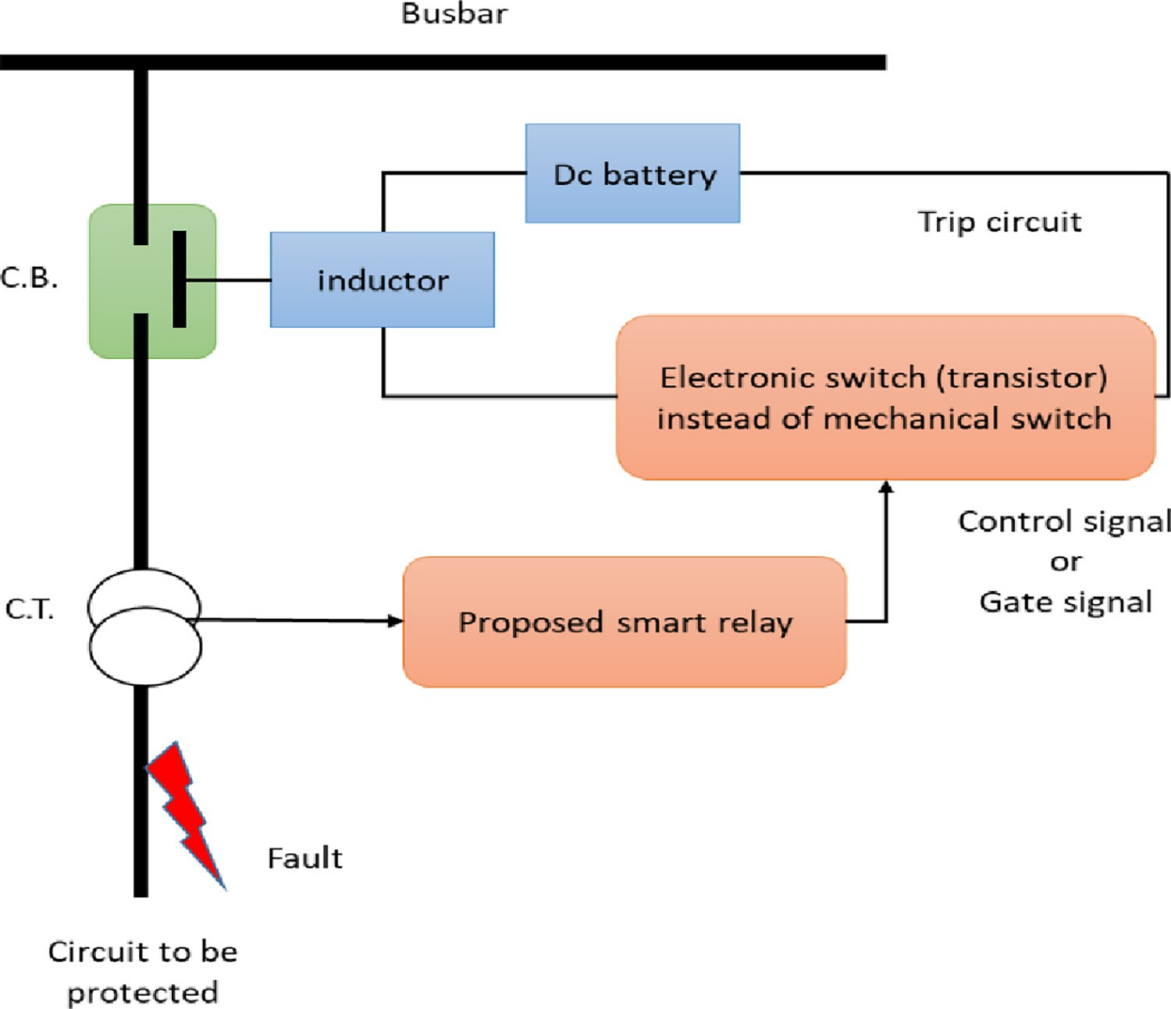
Smart protection system with the proposed adaptive electronic relay.

As previously mentioned, the protection system consists of five components, a circuit breaker, a trip circuit, an instrument transformer, communication channels, and a relay. Fig 2 illustrates a simplified single-phase system with a current transformer or a potential transformer. In earlier versions of protection systems, the relay used contacts to close the trip circuit, functioning via an electro-mechanical switch type based on the principles of electromagnetic attraction or electromagnetic induction.

### 3.1. The proposed adaptive electronic relay

**Fig 3:**
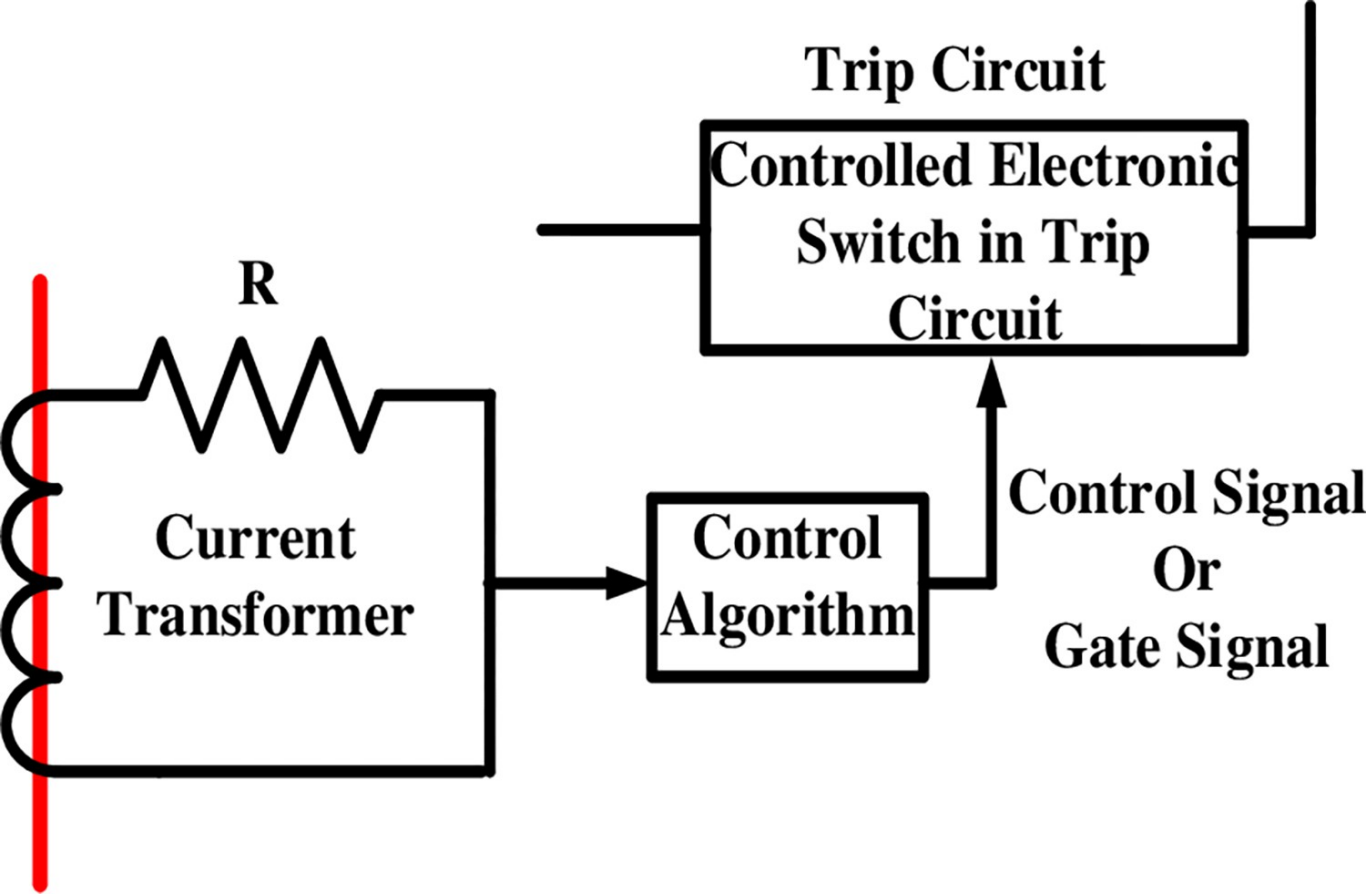
Proposed adaptive electronic relay.

**Fig 4:**
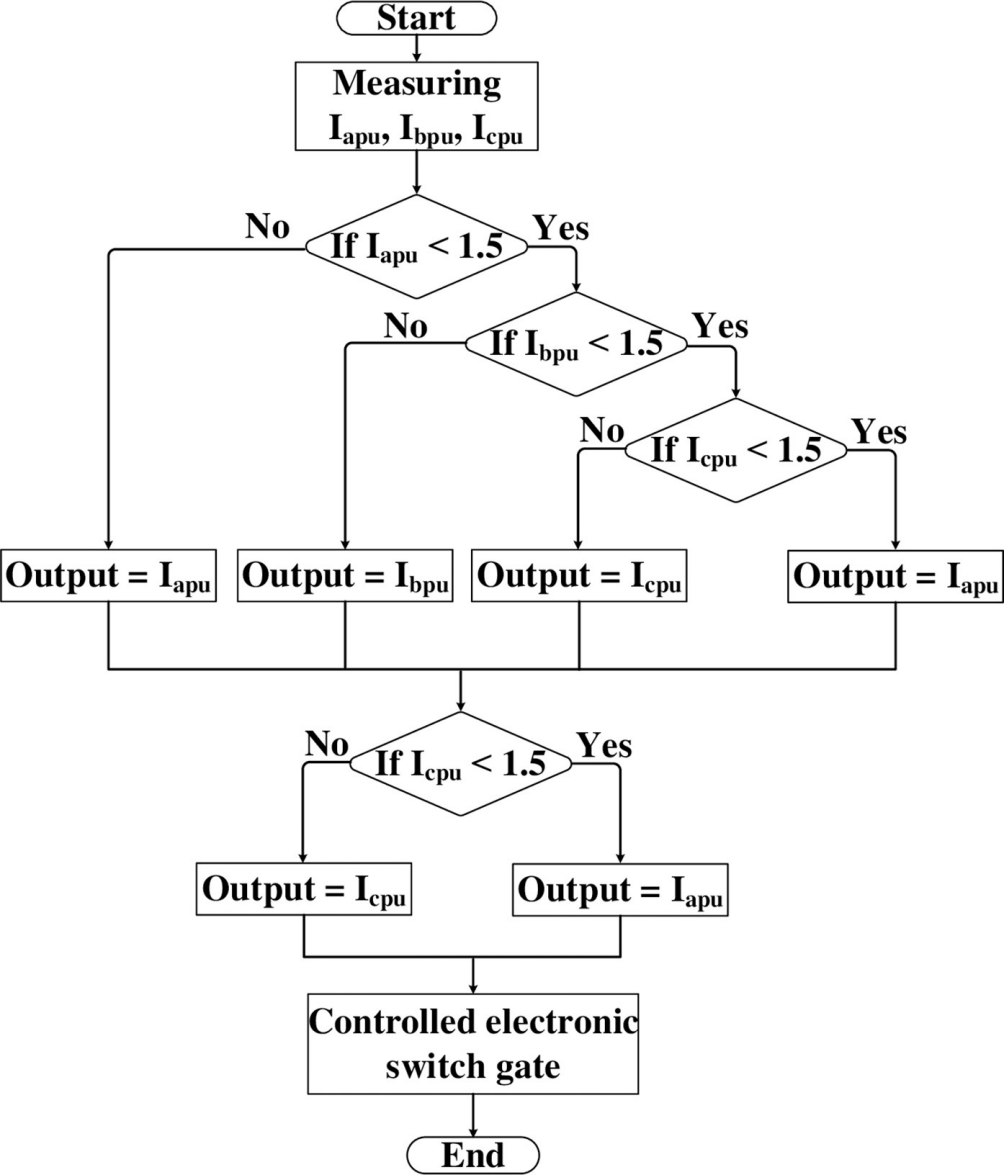
Proposed adaptive electronic relay flowchart.

As shown in Fig 3 (part from Fig 2) the controlled electronic switch in the trip circuit instead of the electro-mechanical switch type has been used. However, the electronic switch is very fast compared to the electro-mechanical switch which makes the system more reliable and has preferable performance. The control signal has been created for the electronic switch using the control algorithm in the microcontroller. The function of the resistor in the relay circuit is to limit the current to a certain value (5A at normal conditions) and it has a known value according to the smart grid and transform ratio. When the control program signals "One," it indicates a closed electronic switch, which in turn closes the trip circuit and opens the circuit breaker. When the program signals “zero,” it means the electronic switch is open, hence the trip circuit is open and the circuit breaker is closed. The program signals one in the case of faults only, whereas it gives zero in normal cases as clarified in the flow chart in Fig 4.

### 3.2. The flowchart of the proposed adaptive electronic relay

The flowchart of the proposed technique for the control algorithm (inside the microcontroller) to regulate the electronic switch is shown in Fig 4. The flowchart comprises three stages. The first stage begins by measuring the per unit values of the three-phase current for the protected device. In the second stage, the per unit current in phase (a) is compared to the allowable value for increased current without causing system issues (1.5 per unit). If phase (a) is within the allowable value, the process checks phase (b), and if phase (b) is also within the allowable value, it proceeds to phase (c). If phases (a), (b), and (c) are all within the allowable value, the system outputs any per unit phase current. However, if any phase exceeds the allowable value during these checks, the process stops and outputs the per unit phase current for that phase. The third stage compares the per unit phase current output from the second stage with the allowable value. If it exceeds the allowable value, a "one" signal is sent, closing the trip circuit and opening the circuit breaker. If it is below the allowable value, a "zero" signal is sent, and the system remains in normal operation.

## 4. Simulation results

The system shown in Fig 1 was created using MATLAB Simulink software. A step-down transformer is used to reduce the grid voltage from 33kV to 11kV. The wind station comprises six turbines, each generating 1.5MW, and is connected to bus 1 via a step-up transformer from 575V to 11kV. The system also includes a PV station supplying 3MW through a step-up transformer from 260V to 11kV. An automatic crowbar system serves as a primary protection system within the distribution network. The system supplies four bus loads, detailed in Table 1, and includes a Variac transformer EV charging station at bus 2, acting as the smart load. Additionally, distributed circuit breakers are integrated throughout the system to protect all elements of the smart grid. To validate the effectiveness of the proposed smart electronic relay across various sections of the smart grid system (transmission or distribution lines, transformer, and load), different faults were simulated.

### 4.1. Fault on the transmission or distribution lines (single phase to ground fault)

**Fig 5:**
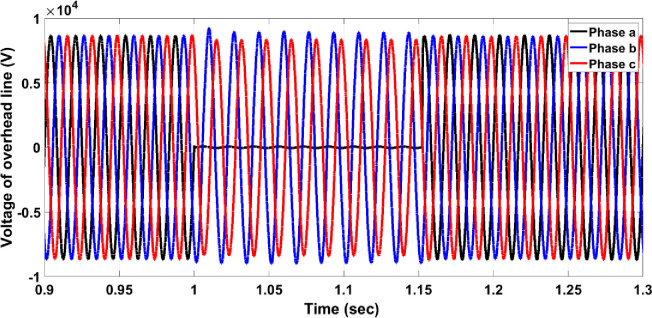
Three-phase voltage with a single line to ground fault without the protection system.

**Fig 6:**
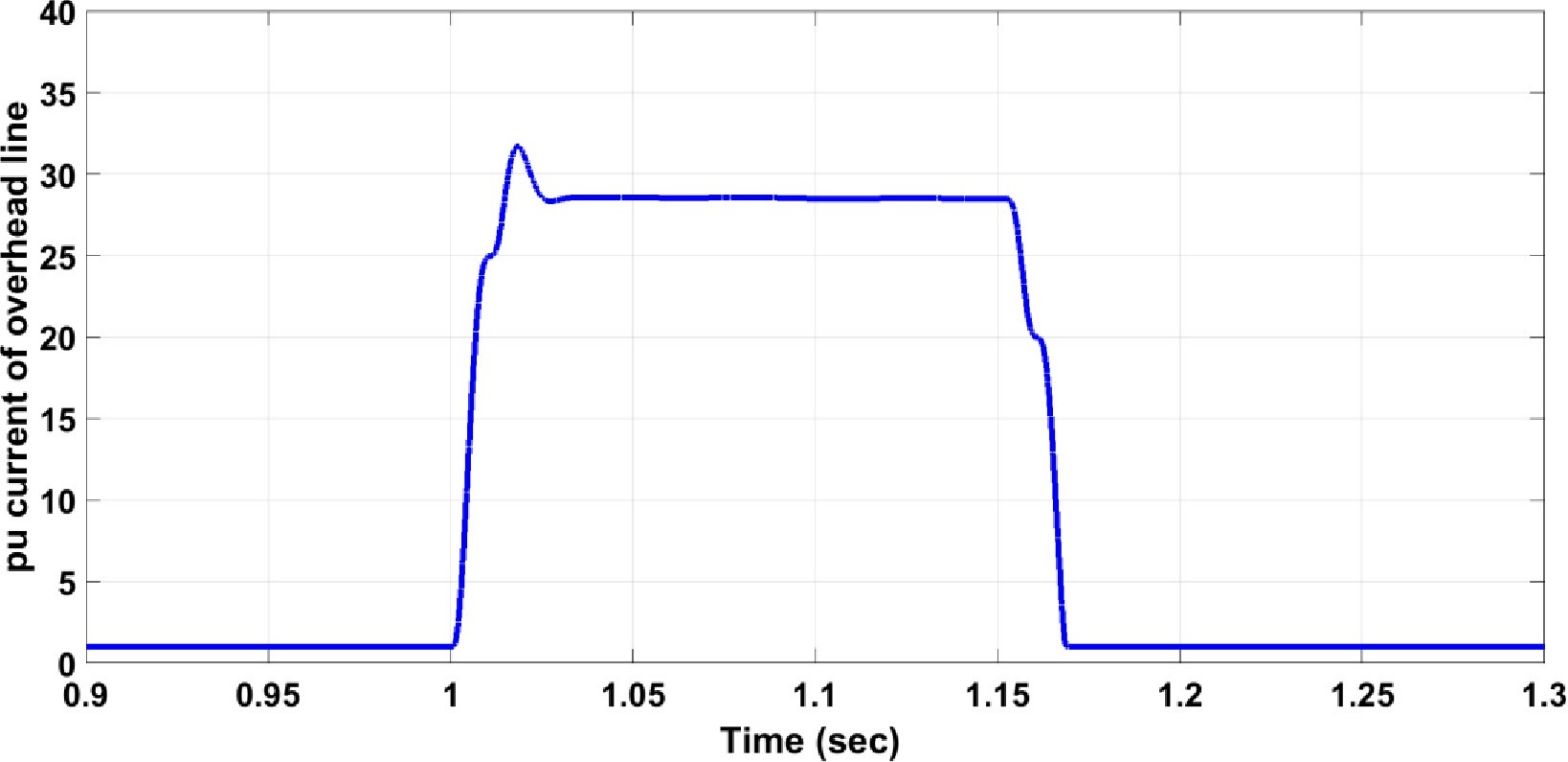
Per unit current of the faulty phase with a single line to ground fault without the protection system.

**Fig 7:**
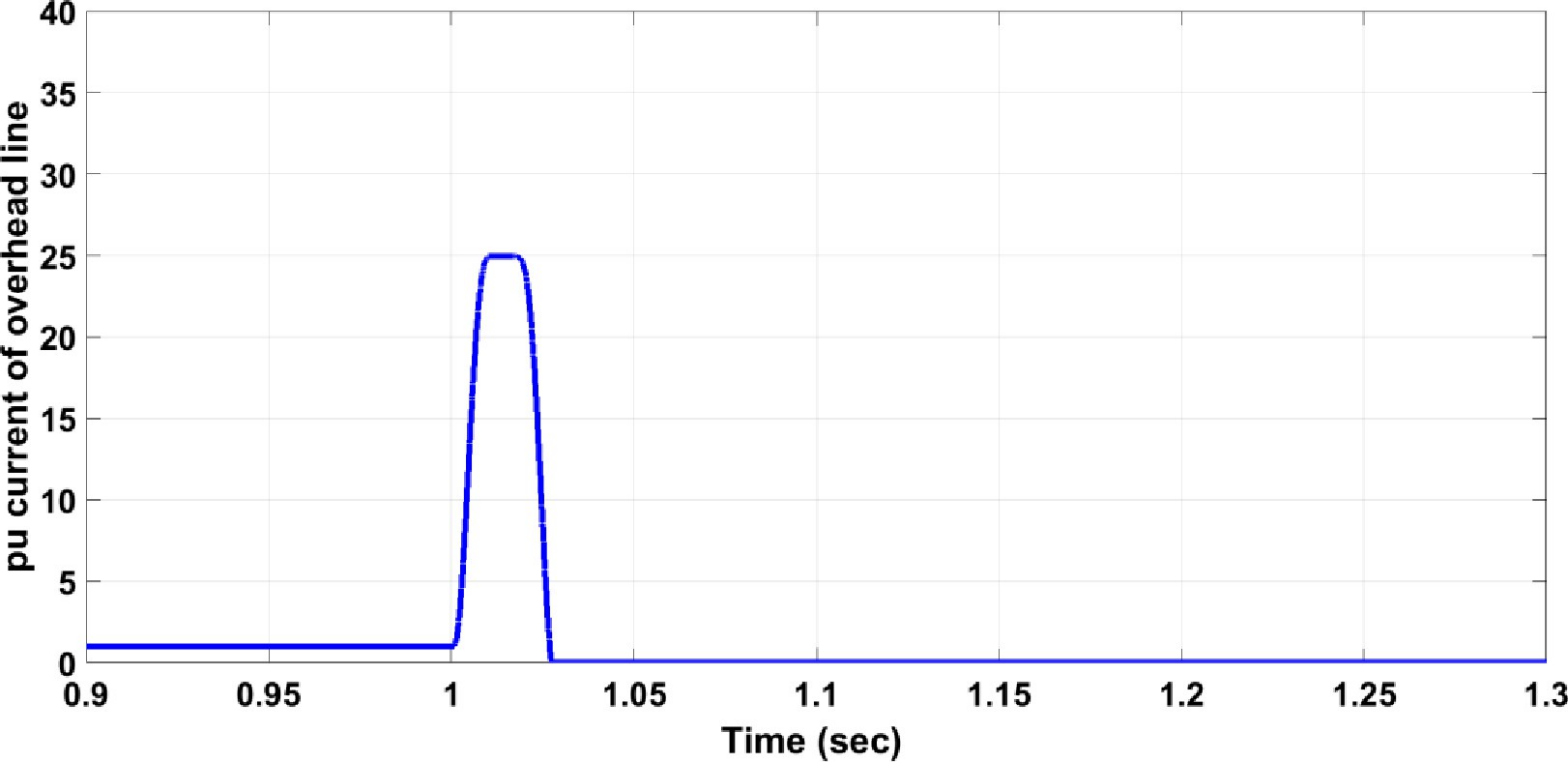
Per unit current of the faulty phase with a single line to ground fault with the protection system.

**Fig 8:**
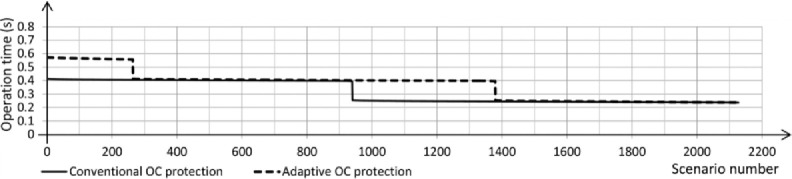
Measured the operating time of conventional and adaptive protection during the phase to earth fault.

The fault on the transmission or distribution lines (overhead lines) commonly occurs in the grid system. The system should be protected against different faults such as single phase to ground fault, the line-line fault, three-line fault, … etc. In this section, single line to ground fault for overhead lines faults has been chosen to be studied due to its widespread among the power systems. If the fault occurs on the overhead line between B4 and B5, circuit breaker 19 must work and separate the faulty part from the rest healthy network system. The three-phase voltage of the overhead lines is disturbed due to the fault as shown in Fig 5; the fault occurred between seconds 1 and 1.15. Fig 6 illustrates the per unit current of the faulty phase without a protection system and it indicates that the value increases to be 28 per unit value which is above the allowable value of 1.5 per unit current. The increase of the current continues during the fault duration (150ms), then, it returns to the normal value after removing the fault. This scenario is dangerous for the network system. Fig 7 shows that the protection system responded swiftly after the per unit current of the faulty phase increased to 25 per unit for 25ms. The 25ms operation period of the circuit breaker is significantly faster compared to the periods mentioned in [3] and [4]. In [3], the operation times of two methods are shown in Fig 8, where the faster method reaches an operation time of 250 ms. The period of 25ms is appropriate for using the automatic crowbar system as the primary protection system beside the proposed system as a backup protection system, due to the automatic crowbar system operating after 10 ms as mentioned in [6]. This increased the reliability of the smart grid.

### 4.2. Fault on the load (exceeded to double magnitude)

**Fig 9:**
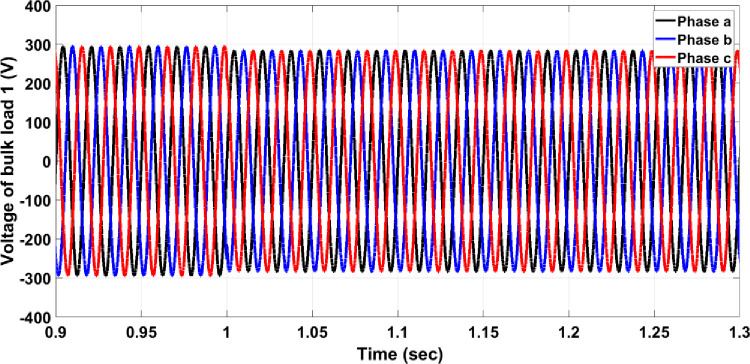
Three-phase voltage without the protection system.

**Fig 10:**
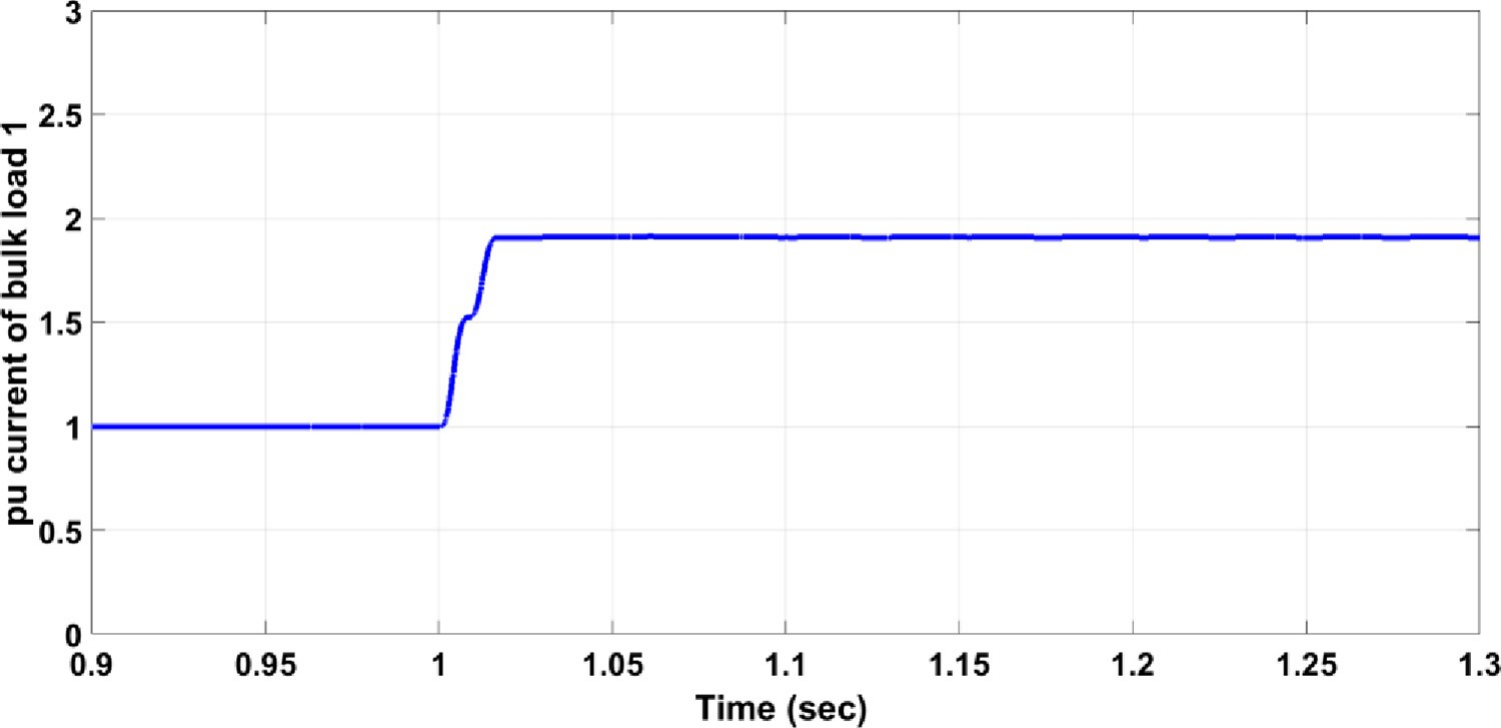
Per unit current of the faulty phase without the protection system.

**Fig 11:**
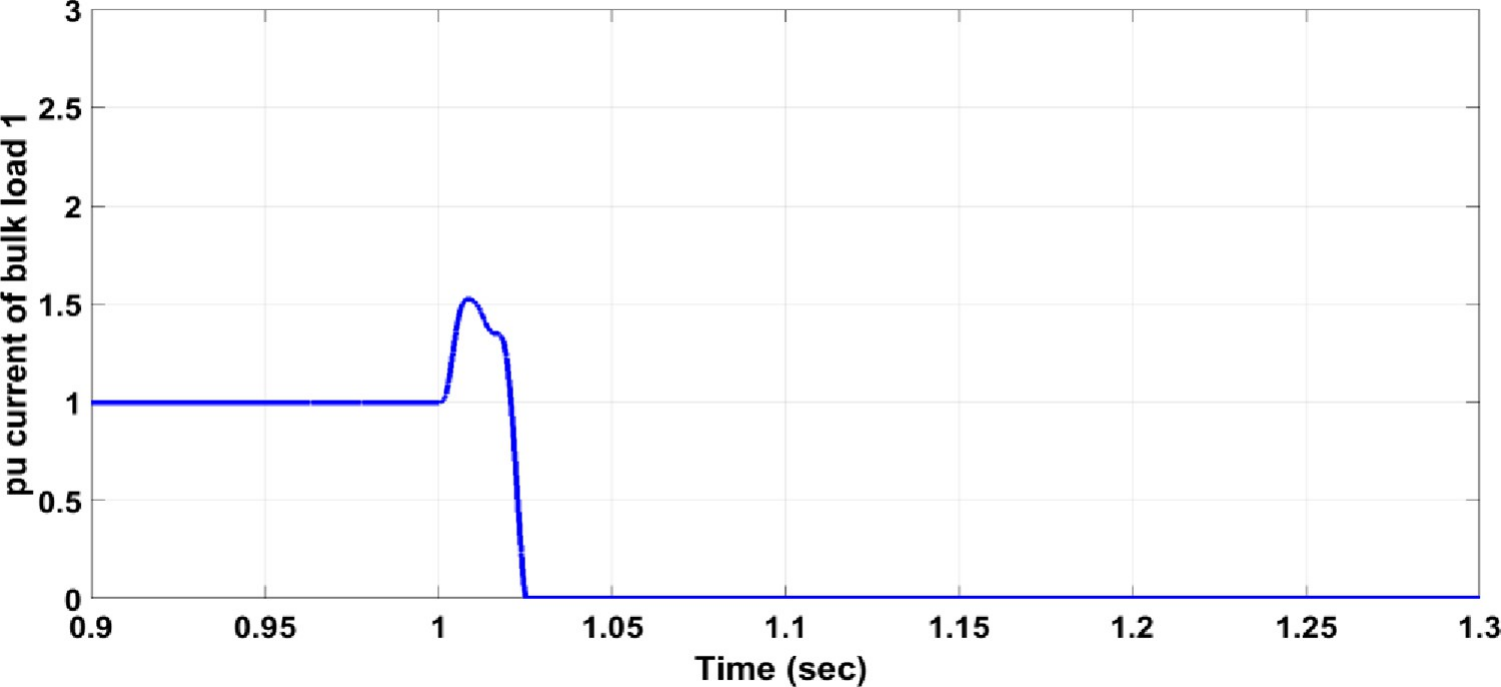
Per unit current of the faulty phase with the protection system.

Load faults can manifest in various forms. In this section, a fault where the load doubles has been examined, causing the current to exceed the allowable value for normal operation. For instance, if Bulk Load 1 doubles, Circuit Breaker 14 must respond to isolate the faulty section from the healthy network. As shown in Fig 9, the three-phase voltage before the load drops from a maximum of 300V to 290V due to the increased load. Without a protection system, the per unit current for the faulty phase rises to 1.9, as illustrated in Fig 10, which is above the allowable value for normal operation. When the protection system is in place, Circuit Breaker 14 activates to separate Bulk Load 1 from the healthy network, as shown in Fig 11. The per unit current of the faulty phase rises to 1.5 and then drops to zero after 25ms, similar to the response observed in overhead line faults.

### 4.3. Fault on the transformer (three-phase short circuit on the secondary winding)

**Fig 12:**
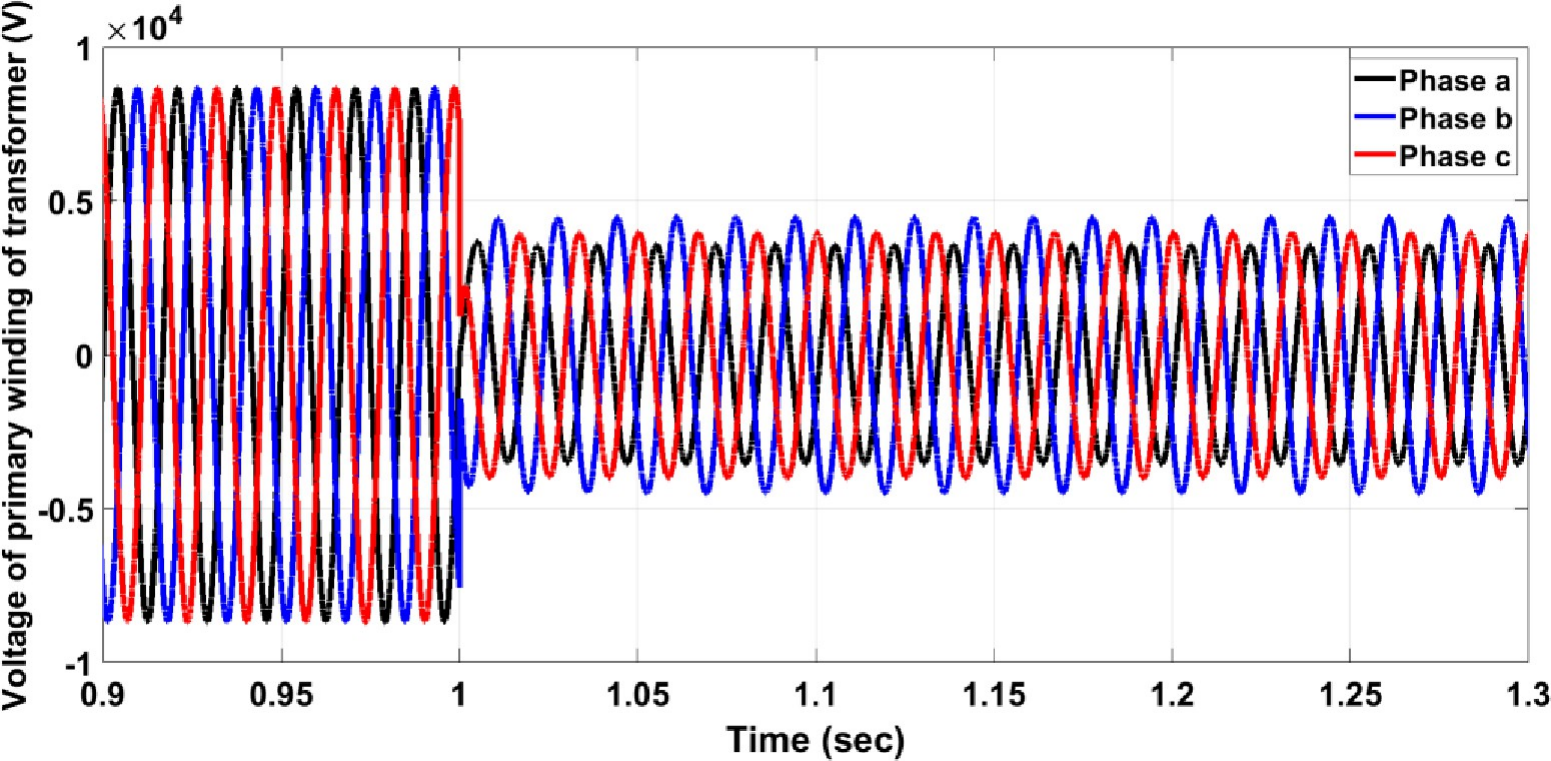
Three-phase voltage of the primary winding transformer without the protection system.

**Fig 13:**
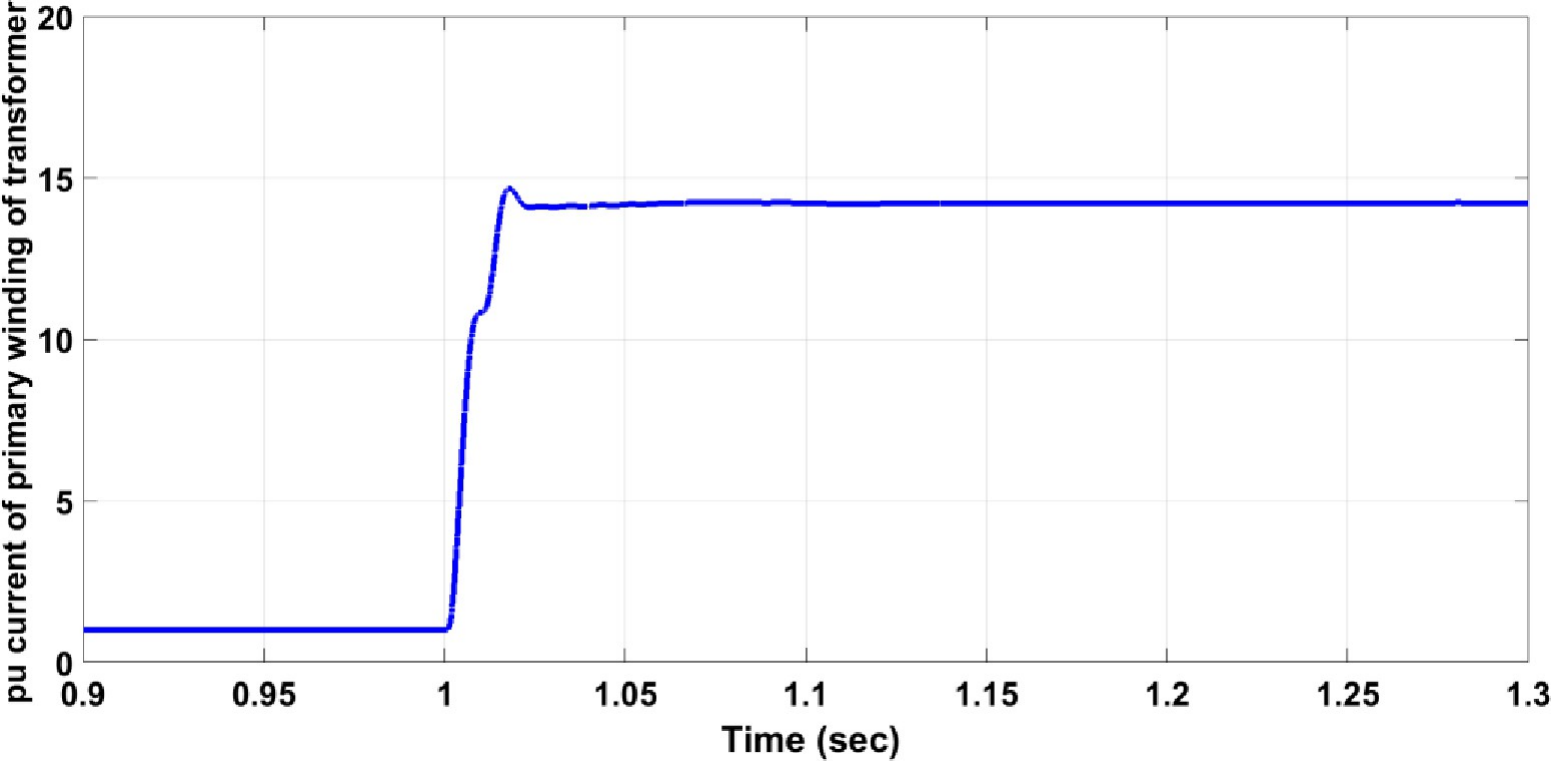
Per unit current of the faulty phase of the primary winding transformer without the protection system.

**Fig 14:**
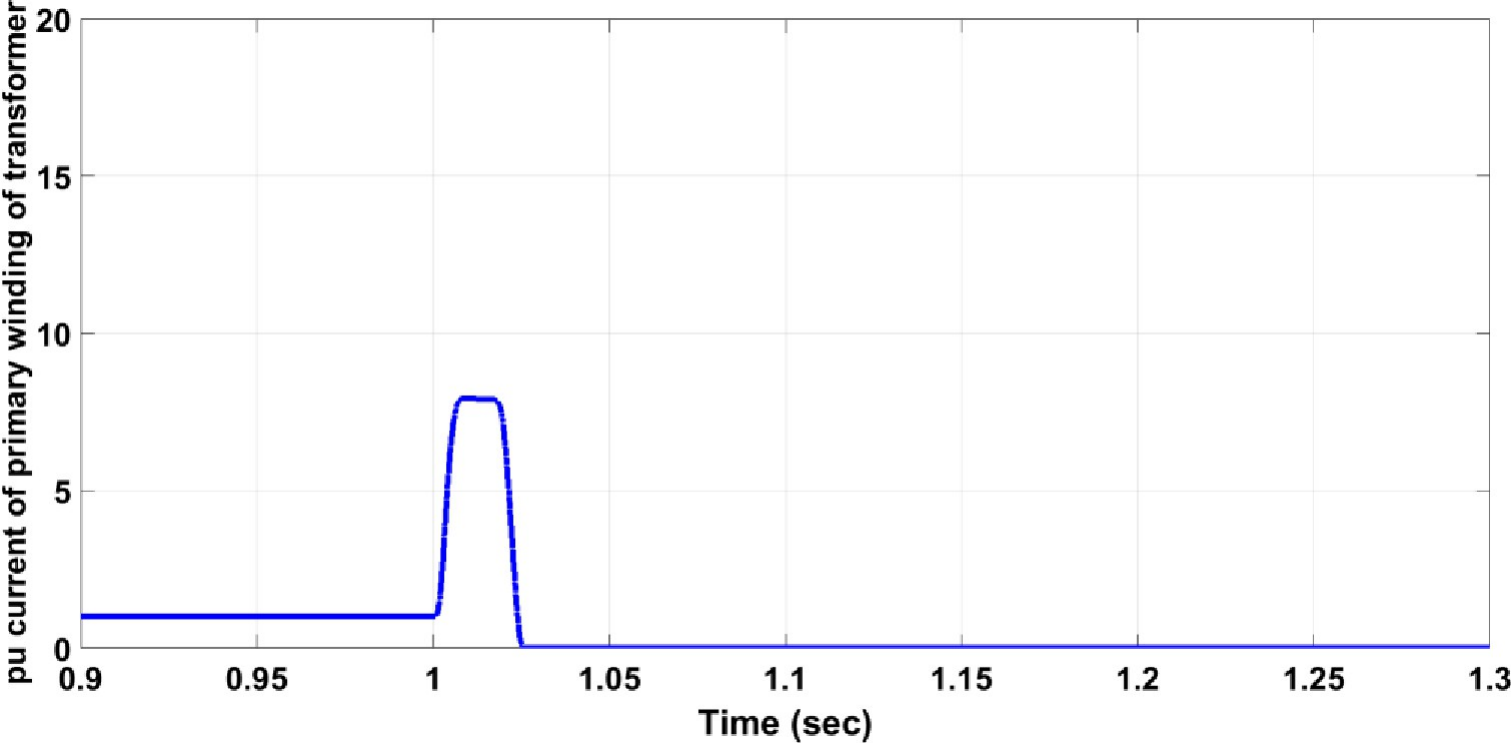
Per unit current of the faulty phase of the primary winding transformer with the protection system.

Transformer faults can occur in various forms, typically as a short circuit between two or three phases on the secondary winding, though any internal fault within the transformer is possible. This section focuses on a three-phase short circuit on the secondary winding of the transformer while measuring the primary winding voltage. For instance, if a fault occurs in the step-down transformer before Bulk Load 2, Circuit Breaker 17 must activate to isolate the faulty section from the healthy network. As shown in Fig 12, the fault significantly affects the primary winding voltage, reducing the maximum value to approximately half and causing disturbances in the symmetrical form. In Fig 13, without the protection system, the per unit current for the faulty phase on the primary winding rises to approximately 14.5, exceeding the allowable value for normal operation. However, with the protection system in place, as shown in Fig 14, the per unit current for the faulty phase increases to around 8 and then decreases to zero after 25 ms, similar to the responses observed in previous cases.

## 5. Comparative study

A comparative study has been mentioned to clarify the effectiveness of the proposed system as illustrated in Table 2. The major components of the comparison are operating time, ease of implementation, relay cost, main limitation, and relay type. It can be noted that the proposed system has achieved the contribution by reaching the minimal operating time compared to the existing systems (25 ms). Moreover, the comparison clarifies the advance of the proposed system in the degree of easy implementation and the relay cost. Existing models face challenges related to operational speed and complexity, while extreme hot weather limits the functionality of the proposed system. The relay type in the proposed model is overcurrent which was used in [3] and [14], while the distance type was used in [15] and [25].

**Table 2:** Comparative study between proposed system and systems in the literature.

Item description	Proposed system	[3]	[14]	[15]	[25]
Operating time (time response)	25ms (buck up protection)	250ms (preferable case)	From 16ms to 100ms	Zone 2 350ms (buck up protection), Zone 3 1000ms (buck up protection)	200ms
Ease of implementation	Easy	More complex	More complex	More complex	More complex
Relay cost	Low	Moderate	Moderate	Moderate	Moderate
Main limitation	Limited ability in high-temperature	Relative Slowness	Complexity	Relative Slowness	Relative Slowness
Relay type	Overcurrent	Overcurrent	Instantaneous overcurrent	Distance	Distance

## 6. Conclusion

This paper presents a proposed adaptive electronic relay designed to convert the system into a smart protection system, achieving key objectives of the smart grid. The effectiveness of the adaptive electronic relay has been validated in this study. In this model, the protection system acts as a backup. The proposed adaptive electronic relay, integrated with the smart grid system, has been validated against faults in overhead lines, loads, and transformers, as detailed in the results. However, its functionality extends beyond these specific faults. Any fault, whether on the generator or any part of the network system, can be swiftly isolated from the healthy system by the adaptive electronic relay, detecting phase current increases beyond allowable thresholds. The system demonstrated excellent performance and rapid fault response, effectively isolating the faulty section from the healthy system. The 25 ms response time of the circuit breaker is sufficient for protecting the system and activating any control mechanisms, such as a controllable crowbar protection system, before the primary protection system engages. The proposed adaptive electronic relay has been tested in multiple conditions such as transmission lines, load, and transformer. The model has shown high reliability and accuracy in all cases which means that the model is applicable and wide-effective.

## 7. Future work and limitation

Future research can explore the interaction between the crowbar, smart fault current controller, and the proposed protection system, examining their roles as both primary and backup protections throughout the smart grid. The proposed adaptive electronic relay, which uses an electronic switch and microcontroller, has limitations due to its inability to withstand high temperatures. Consequently, this relay system may not be suitable for certain locations or applications with high temperatures.

## Abbreviations

GA-PSO-LP: Genetic Algorithm-Particle Swarm Optimization-Linear Programming
AVT: Automatic Variac Transformer
SG: Smart Grig
MG: Micro Grid
DS-DOCR: Dual-Setting Directional Overcurrent Relays
BAP: Busbar Area Protection
DFIG: Doubly-Fed Induction Generator
V, KV, MV: Voltage, Kilovoltage, Megavoltage
Ω: Ohm
PCC: Point ComNon coupling
EV’s: Electric Vehicles
P(W): Power (watt)
K, M: Kilo, Mega
C.B.: Circuit Breaker
C.T.: Current Transformer
sec, ms: Second, millisecond
